# Supplementary material for: Multiple isoforms of the Activin-like receptor baboon differentially regulate proliferation and conversion behaviors of neuroblasts and neuroepithelial cells in the Drosophila larval brain
Source: PLoS One. 2024 Jun 24;19(6):e0305696. doi: 10.1371/journal.pone.0305696 (PMC11195991; doi:10.1371/journal.pone.0305696)

S1 Table

Name	Use	Sequence (5' to 3'); P- indicates phosphorylated
A-indel_f	CRISPR	P-CTTCGCGAACCTTTCGTTACTGGTG
A-indel_r	CRISPR	P-AAACCACCAGTAACGAAAGGTTCGC
B-indel_f	CRISPR	P-CTTCGCAGCCCGTCGTTACACCAGA
B-indel_r	CRISPR	P-AAACTCTGGTGTAACGACGGGCTGC
C-indel_f	CRISPR	P-CTTCGTTGCACGAGGTAGGATCTTC
C-indel_r	CRISPR	P-AAACGAAGATCCTACCTCGTGCAAC
A-indel_for	PCR, seq	AGCAGCAACCAACAACCAAT
A-indel_rev	PCR, seq	GCTAAGGGGAAAACTGAGGC
B-indel_for	PCR, seq	AACGGACGGCTTTTGTTTCA
B-indel_rev	PCR, seq	CGTGCGAGTGAACAGAGAAA
C-indel_for	PCR, seq	TGGTAACCAAAGCTGCTTCG
C-indel_rev	PCR, seq	TGTGCAAGGTGTGTGTTAGC

S1 Figure

Exon 4A, portion of WT sequence near guide site

```
GAA TGC CTC ACC AGT AAC GAA AGG TTC
E.. C.. L.. T.. S.. N.. E.. R.. F..
```

A-indel (*AΔ7*) AM3A AJP511 Δ7f2 (run on then stop within 4A)

```
GAA TGC CTA ACG AAA GGT
E.. C.. L.. T.. K.. G.. S.. I.. R.. I.. etc
```

A-indel (*AΔ9*) AM22C AJP510 Δ9^f1

```
GAA TGG TTC GAA AGG TTC GAT ACG TAT AGG ATT GAT TGC
E.. W.. F.. E.. R.. F.. D.. T.. Y.. R.. I.. D.. C..
```

Exon 4B, portion of WT sequence near guide site

```
CGC TCC ATC TGG TGT AAC GAC GGG CTG
R.. S.. I.. W.. C.. N.. D.. G.. L..
```

B-indel (*BΔ4*) A40A AJP480 Δ4^stop (IW->M*)

```
CGC TCC ATG TAA
R.. S.. M.. *
```

B-indel (*BΔ1*) B29C AJP483 Δ1f2 (run on with no stop within 4B)

```
CGC TCC ATC TGT GTA ACG ACG GGC TGC
R.. S.. I.. C.. V.. T.. T.. G.. C.. etc
```

Exon 4C, portion of WT sequence near guide site

```
TTG CCA CCT GAA GAT CCT ACC TCG TGC
L.. P.. P.. E.. D.. P.. T.. S.. C..
```

C-indel (CΔ1) CM9B AJP522 Δ1^f2 (run on with stop in 4C)

```
TTG CCA CCT GAA TGC CTA CCT CGT GCA
L.. P.. P.. E.. C.. L.. P.. R.. A.. etc
```

C-indel (*CΔ9*) CM7C AJP520 Δ9f1

```
TTG CCA CCT ACC TCG TGC AAG
L.. P.. P.. T.. S.. C.. K.. etc
```

S2 Figure

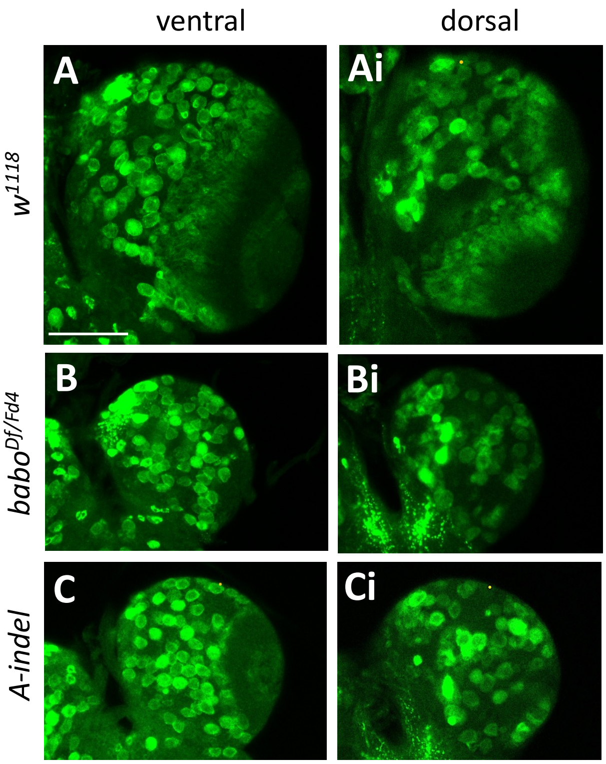

S3 Figure

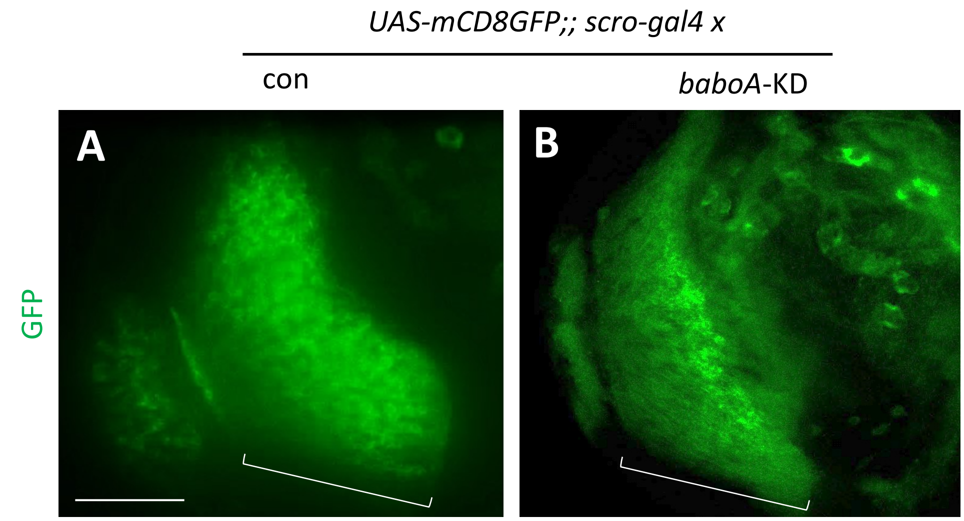

S4 Figure

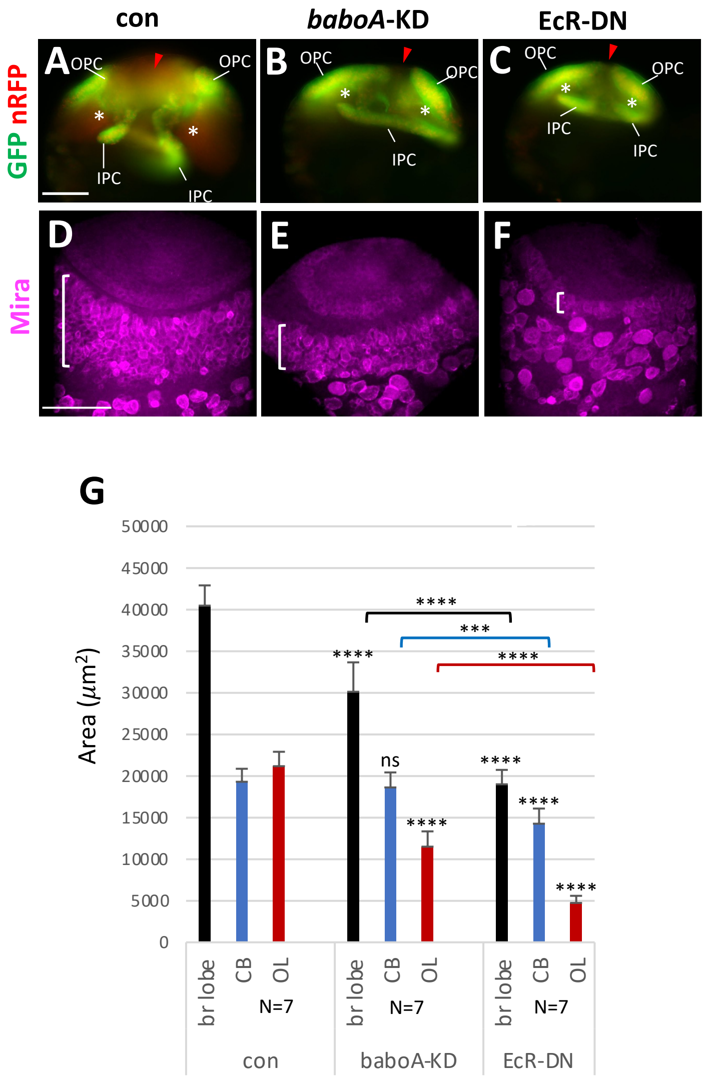

con
baboA-KD
EcR-DN
GFP nRFP
A
B
C
OPC
IPC
Mira
D
E
F
G
Area (μm²)
50000
45000
40000
35000
30000
25000
20000
15000
10000
5000
0
****
***
****
****
ns
****
****
****
****
br lobe
CB
OL
N=7
con
baboA-KD
EcR-DN

S5 Figure

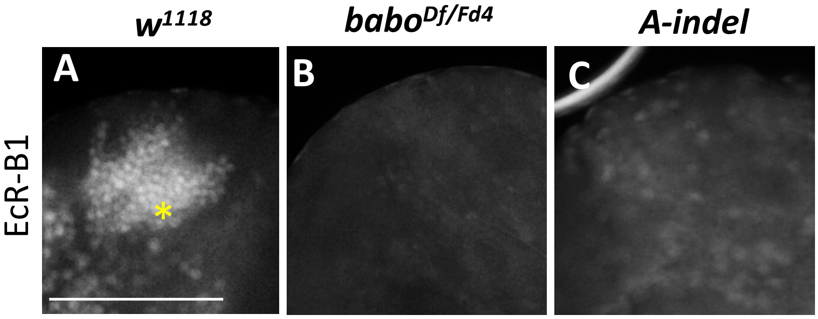

Supplement: S1 File — (PDF) [file pone.0305696.s001.pdf]
